# Supplementary material for: Study-Related Work and Commuting Accidents among Students at the University of Mainz from 12/2012 to 12/2018: Identification of Potential Risk Groups and Implications for Prevention
Source: Int J Environ Res Public Health. 2020 May 23;17(10):3676. doi: 10.3390/ijerph17103676 (PMC7277531; doi:10.3390/ijerph17103676)
Supplement: Supplementary file 1 [file ijerph-17-03676-s001.pdf]

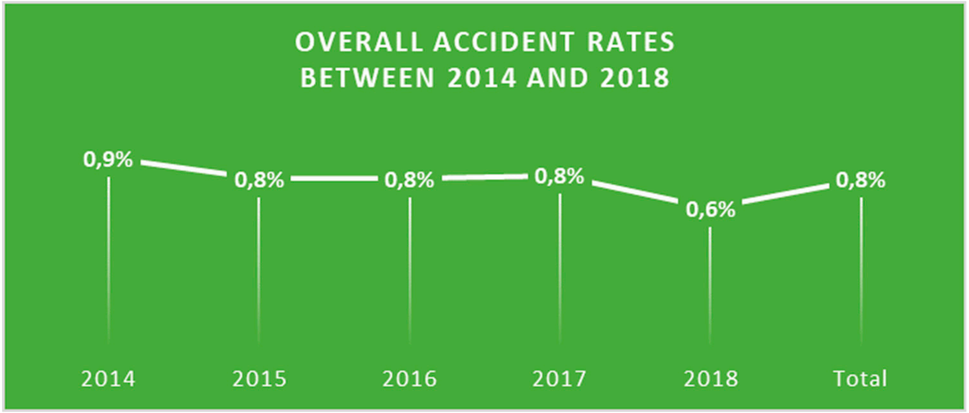

Figure S1. Overall Accident Rates Between 2014 and 2018.

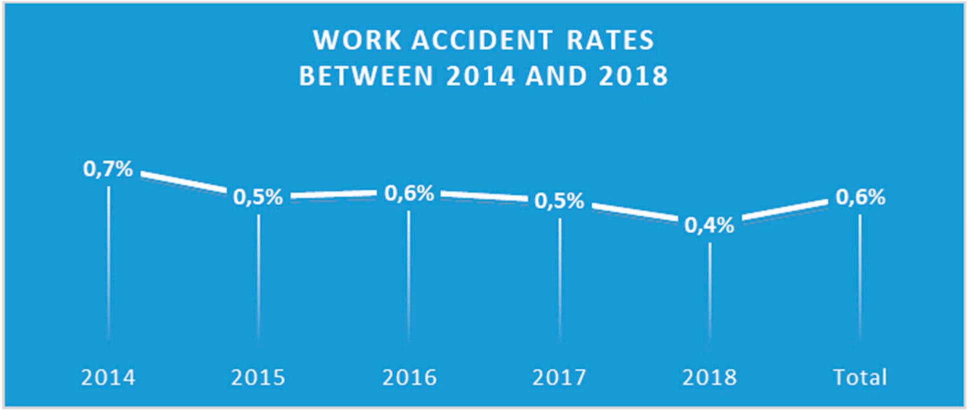

Figure S2. Work Accident Rates Between 2014 and 2018.

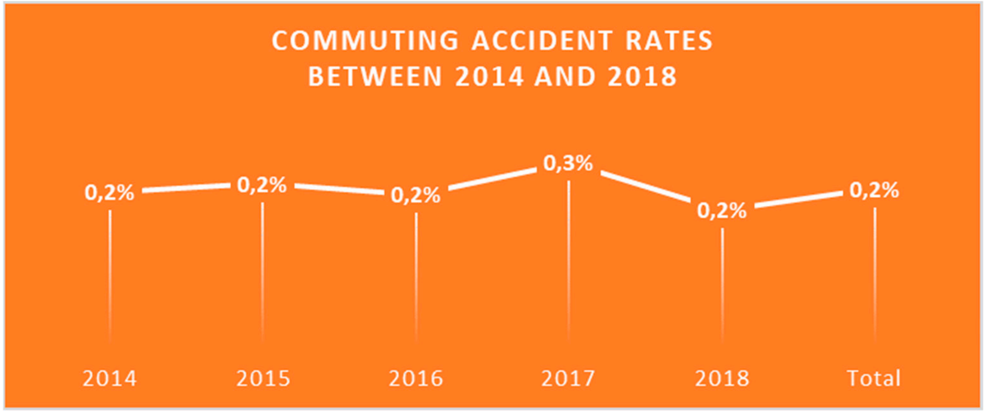

Figure 3. Commuting Accident Rates Between 2014 and 2018.

Table S1. Description of the basic population according to the annually published Data and Statistics Report of the JGU: total number of registered students at the JGU per faculty/school (*N*) and percentage of female students in the winter terms 2010/11 until 2018/19.

	**Winter term 2012/13**	**Winter term 2013/14**	**Winter term 2014/15**	**Winter term 2015/16**	**Winter term 2016/17**	**Winter term 2017/18**	**Winter term 2018/19**
**Faculty / School**	***N* (% females)**	***N* (% females)**	***N* (% females)**	***N* (% females)**	***N* (% females)**	***N* (% females)**	***N* (% females)**
F 01 – Catholic and Evangelic Theology	581 (49)	545 (52)	495 (55)	466 (55)	465 (55)	411 (55)	340 (54)
F 02 – Social Sciences, Media and Sport	5,929 (61)	5,776 (63)	5,596 (64)	5,125 (65)	5,000 (66)	5,053 (65)	5,096 (64)
F 03 – Law and Economic Sciences	5,730 (51)	5,686 (51)	5,761 51	5,755 (52)	5,778 (52)	6,058 (53)	6,006 (53)
F 04 – Medicine	3,510 (62)	3,542 (62)	3,524 (63)	3,616 (62)	3,721 (62)	3,832 (63)	3,885 (63)
F 05 – Philosophy and Philology	7,108 (72)	6,930 (71)	6,416 (71)	5,760 (73)	5,770 (73)	5,705 (72)	5,672 (72)
F 06 – Translation, Linguistic and Cultural Sciences	1,868 (79)	1,798 (80)	1,732 (80)	1,636 (78)	1,587 (79)	1,490 (80)	1,383 (80)
F 07 – History and Culture Sciences	2,875 (56)	2,845 (57)	2,593 (57)	2,212 (57)	2,063 (58)	1,935 (58)	1,837 (57
F 08 – Physics, Mathematics and Computer Sciences	2,518 (33)	2,519 (31)	2,530 (31)	2,526 (31)	2,408 (29)	2,362 (30)	2,367 (30)
F 09 – Chemistry, Pharmaceutical Sciences, Geography and Geosciences	3,838 (46)	3,897 (46)	3,817 (47)	3,738 (48)	3,557 (48)	3,424 (48)	3,085 (49)
F 10 – Biology	1,830 (63)	1,805 (62)	1,725 (63)	1,649 (63)	1,549 (65)	1,497 (66)	1,493 (65)
School of Music Mainz	339 (52)	357 (52)	350 (53)	344 (52)	353 (52)	323 (52)	338 (52)
School of Art Mainz	147 (73)	159 (74)	154 (73)	143 (76)	149 (79)	134 (81)	138 (75)
International Preparatory and Language Center	167 (61)	169 (73)	258 (65)	162 (65)	267 (55)	302 (53)	327 (51)
Total	36,440 (59)	36,028 (59)	34,951 (59)	33,132 (59)	32,667 (59)	32,526 (59)	31,967 (59)

F, faculty; JGU, Johannes Gutenberg University. The *International Preparatory and Language Center* exists since October 2012. It is a fusion of the formerly separate central institutions *Language Center* and *Preparatory College.*

Table S2. Specific types of and reasons for study-related commuting (*N* = 363) and work accidents (*N* = 922).

	N (percentage)	Reason for accident	*N* (percentage)
Commuting accidents			
Injured by fall	199 (54.8%)	Fell/tripped over something	101 (50.1)
		Twisted one`s ankle	56 (28.1)
		Slipped down	39 (19.6)
		Others	3 (1.5)
Injured by bump/hit	149 (41.0%)	Collided with someone/something	118 (79.2)
		Banged on something	24 (16.1)
		Others	7 (4.8)
Others	9 (2.5%)	---	---
Not reported	6 (1.7%)	---	---
Work accidents			
Injured by fall	344 (37.3%)	Twisted one`s ankle	171 (49.7)
		Fell/tripped over something	81 (23.5)
		Miscarried rotation	53 (15.4)
		Slipped down	25 (7.3)
		Others	14 (4.0)
Injured by bump/hit	206 (22.3%)	Hit by something	97 (47.1)
		Banged on something	50 (24.3)
		Collided with someone/something	49 (23.8)
		Others	10 (4.9)
Injured by mechanical impact	204 (22.1%)	Pricked oneself on something/pricked by someone	120 (58.8)
		Cut oneself with/on something	58 (28.4)
		Others	26 (12.8)
Injured by physical, chemical or biological impact	75 (8.1%)	Injured by hazardous substance	55 (73.3)
		Burned/scalded oneself on something	12 (16.0)
		Others	8 (10.7)
Others	33 (3.6%)	---	---
Not reported	60 (6.5%)	---	---
